# A New Method for Traffic Participant Recognition Using Doppler Radar Signature and Convolutional Neural Networks

**DOI:** 10.3390/s24123832

**Published:** 2024-06-13

**Authors:** Błażej Ślesicki, Anna Ślesicka

**Affiliations:** 1 Department of Avionics and Control Systems, Faculty of Aviation Division, Polish Air Force University, 08-521 Dęblin, Poland; 2 Institute of Navigation, Polish Air Force University, 08-521 Dęblin, Poland; a.slesicka@law.mil.pl

**Keywords:** Doppler radar, artificial intelligence, deep learning, convolution neural network, micro-Doppler signature

## Abstract

The latest survey results show an increase in accidents on the roads involving pedestrians and cyclists. The reasons for such situations are many, the fault actually lies on both sides. Equipping vehicles, especially autonomous vehicles, with frequency-modulated continuous-wave (FMCW) radar and dedicated algorithms for analyzing signals in the time–frequency domain as well as algorithms for recognizing objects in radar imaging through deep neural networks can positively affect safety. This paper presents a method for recognizing and distinguishing a group of objects based on radar signatures of objects and a special convolutional neural network structure. The proposed approach is based on a database of radar signatures generated on pedestrian, cyclist, and car models in a Matlab environment. The obtained results of simulations and positive tests provide a basis for the application of the system in many sectors and areas of the economy. Innovative aspects of the work include the method of discriminating between multiple objects on a single radar signature, the dedicated architecture of the convolutional neural network, and the use of a method of generating a custom input database.

## 1. Introduction

In recent years, it is important to note the tremendous growth of interest in the application of artificial intelligence in various areas of life. In particular, this phenomenon concerns the development of deep learning algorithms [1]. Deep learning refers to multilayer neural networks, which simultaneously perform two functions. The first concerns the generator of diagnostic features for the process under analysis. The second performs the function of a classifier or regression system. This approach creates a great tool to replace humans, first of all, in the difficult field of describing a process with specialized descriptors, the creation of which requires great expert skills. Consequently, this approach to the non-interventional method of feature generation is much more effective than the descriptor generation methods traditionally used. In particular, it improves the accuracy of system performance, and for this reason, deep network technology has recently rapidly become one of the most popular areas in computer science [2].

Fukushima’s multilayer neocognitron, defined in the early 1990s [3], can be considered the creator of these networks. The real development of these networks is in fact attributable to Professor LeCun [4], who defined the basic structure and learning algorithm of a specialized multilayer network, called a convolutional neural network (CNN). A turning point in the development of deep neural networks came in 2012 when Alex Krizhevsky’s team won the ImageNet competition in image recognition by several lengths over other teams [5].

Currently, CNN is the basic structure used on a large scale in image processing. Meanwhile, many varieties of networks have been developed as modifications of the basic CNN structure (e.g., UNN), as well as networks that differ fundamentally from CNNs. Examples include the autoencoder (AE) as a multilayer nonlinear generalization of the linear PCA network [2], recurrent networks of the Long Short-Term Memory (LSTM) type [1]—which is an effective solution to the problem of back-propagation over time—or the Restricted (multilayer) Boltzmann Machine (RBM) used in the Deep Belief Network (DBN) [6].

Recently, there has been considerable interest in using radar data as input to deep neural networks [7]. Radar as a sensor is an active device and its use is independent of atmospheric conditions since microwaves are able to penetrate the atmosphere under virtually any conditions. Hence, it is possible to use radar, for example, SAR to image the terrain both during the day and night, as well as in the presence of clouds, fog, and smoke. In addition, radar as a sensor has a much longer range than optoelectronic sensors like cameras or lidar. In addition, radar has the ability to detect moving objects, and is even capable of detecting objects masked by, for example, vegetation or leaves. Unfortunately, radar imagery is difficult to interpret, requiring skilled analysts [8]; hence, the authors propose a solution in this article to replace the difficult analysis of radar imagery by using deep neural networks to distinguish the types of objects moving opposite the radar. From a military point of view, it should also be added that radar as an active sensor is thus easy to detect by the enemy [9].

The use of the Doppler phenomenon in radiolocation is well known. On the other hand, the description of the occurrence of the micro-Doppler effect is noteworthy [10]. The main difference between the micro-Doppler effect and the classical Doppler effect is that the Doppler effect measures the change in frequency of a wave associated with the motion of an object, while the micro-Doppler effect additionally analyzes the frequency changes resulting from internal structural movements of an object. In other words, while the Doppler effect deals with the usual frequency changes caused by the motion of the object as a whole, the micro-Doppler effect additionally studies the microscopic movements within the object’s structure that also affect the wave [11]. In the case of radar, this phenomenon occurs when the radar-radiated object of interest performs rotation or mechanical vibration. Then, in the received signal, the described vibrations or movements in the object’s body axis can induce additional frequency components in the echo signal. Examples of movements generating the micro-Doppler phenomenon are the rotors of helicopters (propellers of a fixed-wing aircraft, the engine compressor and blade assemblies of a jet aircraft) or the limbs of a person while walking [12]. In summary, the movements of various parts of an object placed in front of the radar produce micro-Doppler signatures that can be used to identify the object.

The authors of this article are very clear about the novel content of the method of identifying objects using FMCW radar, processing the obtained signals using the short-time Fourier transform (STFT), and then the obtained spectrograms using deep neural networks. The proposed solution eliminates the known limitations of existing techniques for counting (distinguishing) people, cyclists, or cars with the help of a camera in vision systems since the camera requires a clear line of sight, while the quality of the imaging obtained with the camera depends on the lighting conditions.

These things considered, the application of the presented solution to automotive radar—in addition to its essential ability to measure the distance and relative speed of objects in front of cars—can serve to improve the driver’s ability to perceive objects in poor visibility or objects in blind spots.

Taking the above into account, including in particular the recalled properties of radar relative to other imaging sensors, by collecting radar signatures in real-time and using machine learning algorithms to detect pedestrians, cyclists, and cars, it is possible to develop a system to warn a driver or other road users of the possibility of a collision with a pedestrian at night. Such a system could have applications in autonomous vehicles, in which a signal can be developed to activate the automatic braking system (ABS) when necessary.

The proposed system can be integrated into the vehicle’s intelligent system, which provides real-time pedestrian detection and warning mechanisms by vibrating the steering wheel and displaying a message on the monitor/dashboard to warn the driver of a collision with a pedestrian.

The system can also be extended to detect and avoid cyclists and animals and can be implemented in autonomous vehicles to support automatic braking systems.

In addition, the system of automatic detection of people, animals, or vehicles can be used in sectors of the economy such as production halls where accidental uncontrolled interference by a person or other object can disrupt the normal operation of the enterprise or expose people, including employees, to loss of life or health.

Innovative solutions in this article also include the development of a method for the generation and flow of simulated radar data.

The rest of this paper is organized as follows. Section 2 describes the state of the art of the issues addressed. Section 3 describes the method of radar data generation as input data for a special convolutional neural network structure. In addition, a brief characterization of the FMCW radar, object models, Fourier transform (SFTF), and a description of the construction and parameters of convolutional neural networks is also included. Section 4 presents the results of the experiments. Finally, Section 5 presents conclusions and discussions of the obtained results.

## 2. Related Works

Target detection and classification based on deep learning from micro-Doppler signatures is gaining popularity in the field of automatic recognition of moving objects on the ground, such as people, animals, or vehicles. Micro-Doppler signatures are characteristic frequency patterns of radar signals that result from an object’s motion. Through deep learning, models can be trained to analyze these signatures, making it possible to automatically recognize and classify different types of objects based on their motion [13].

In the paper [14], a MAFAT dataset containing echo signals from humans and animals collected by different Doppler radars in different locations, terrains, and with different SNRs was used to train a six-layer convolutional neural network. To achieve higher classification accuracy, the data were further augmented by randomly shifting frequency/time, adding noise, and inverting the image vertically/horizontally.

On the other hand, a paper [15] proposed a CNN consisting of five dense blocks (i.e., 3 × 3 convolutional layer followed by 1 × 1 convolutional layer) and five transition blocks (i.e., 1 × 1 convolutional layer followed by 2 × 2 pooling layer) for human motion classification based on micro-Doppler signatures. The performance of this network was tested on two datasets containing echoes associated with six human movements (walking, running, crawling, forward jumping, and boxing), obtained by simulation and measurement, respectively. The main feature of the human-motion-recognition algorithm in [15] is that the proposed network is more robust to the variable aspect of the target angle than most classical CNN models, such as VGGNet, ResNet, and Dense-Net.

In articles [16,17,18], the authors focused on the problem of recognizing single individuals/groups/vehicles based on micro-Doppler signatures using deep learning (DL). To improve the training efficiency of the network, pre-trained classical CNN models, such as VGG16, VGG19, and AlexNet, and a learning-transfer technique were used. Human target/vehicle tracking data collected from Ku-band pulsed Doppler radar (RadEch) included various typical scenarios, such as a single person, a group walking, running, and truck movement. These data were used to evaluate the performance of the proposed network. In addition, data augmentation was applied, meaning that the training data were augmented by vertically inverting the image and circularly shifting it to compensate for the limited amount of training data available. Data augmentation is a technique used in machine learning that involves generating new training examples by making changes to existing data to increase the variety and quantity of training data.

The works [19,20] used pre-trained classical CNN models (for example, GoogLeNet) for drone classification. In particular, in work [19], micro-Doppler signatures and velocity-cadence diagrams obtained by 14 GHz FMCW radar in indoor and outdoor experiments were combined as Doppler images, based on which drones with different numbers of engines were classified. In [20], both a pre-trained GoogLeNet and a deep CNN series with 34 layers were used to classify drones/birds at night. The RGB and the grayscale echo signal dataset collected by 24 GHz and 94 GHz FMCW radars are used to train the two networks, respectively. One distinctive feature of the networks presented in [20] is that clutter and noise have been treated as two separate subclasses.

In [21,22], the authors proposed a two-layer deep network having symmetric connections between nodes (neurons) of these layers (DBN, Deep Belief Networks). This network is built by juxtaposing classical RBM and RBM with Gauss-Bernoulli distribution (GBRBM), which is similar to the solution proposed in [19] to solve the problem of microdrone detection and classification. The classification was based on the Doppler signatures of the objects in question and their spectral signature patterns (SCF) (i.e., the Fourier transform of the autocorrelation function). The performance of the proposed DBN network was tested on echo signals collected from three microdrones using S-band Doppler radar.

In conclusion, it is worth noting that a comprehensive overview of the application of algorithms based on deep learning (DL), such as convolutional neural networks (CNNs) for detecting and classifying different types of objects, was presented in [23]. This paper discusses the application areas of deep learning, i.e., radar waveform and antenna array design; radar waveform recognition of passive or low-probability-of-detection (LPI) radars; automatic target recognition (ATR) based on high-resolution range profiles (HRRPs); Doppler signatures and synthetic-aperture radar (SAR) images; and radar interference/noise recognition and suppression.

Taking into account the above literature review, the authors of this article proposed a missing approach based on simulated data generated in the Matlab environment and a novel method of identifying objects using FMCW radar operating at 77 GHz, processing the obtained signals using STFT, and then the obtained spectrograms using a specialized deep neural network structure. In addition, the Table 1 below contains a list of acronyms to facilitate reading and analysis of the article.

## 3. Method

**Figure 1:**
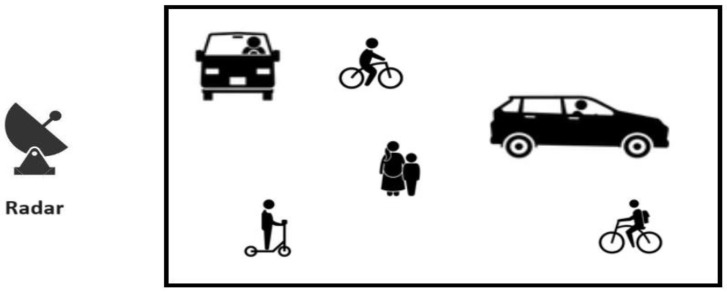
Area of interest of radar.

This paragraph describes the radar, pedestrian, cyclist, and car models adopted in the article. A frequency-modulated continuous-wave (FMCW) radar operating at *f_C_* = 77 GHz was adopted. The Figure 1 below shows a view of a simulated situation, where the radar will radiate a specific area where there may be moving pedestrians, cyclists, and cars.

Such a situation can occur on roads, in parking lots, airports, or other important public places, where it is necessary to take care of the safety or identification of emerging objects.

### 3.1. FMCW Radar

Frequency-modulated continuous-wave radar allows the detection and estimation of distances to objects within the radar beam. The principle of such radar is to probe the space with a signal with linear frequency modulation. The echo signal is then subjected to spectral analysis. The position of the striations (exceeding the assumed detection threshold) on the frequency axis is directly proportional to the distance between the radar antenna and the observed spatial objects. The advantages of such a radar are a simple and uncomplicated design, low power of the probing signal, and, thus, what is important from the military point of view—a low susceptibility to detection by the enemy [24].

**Figure 2:**
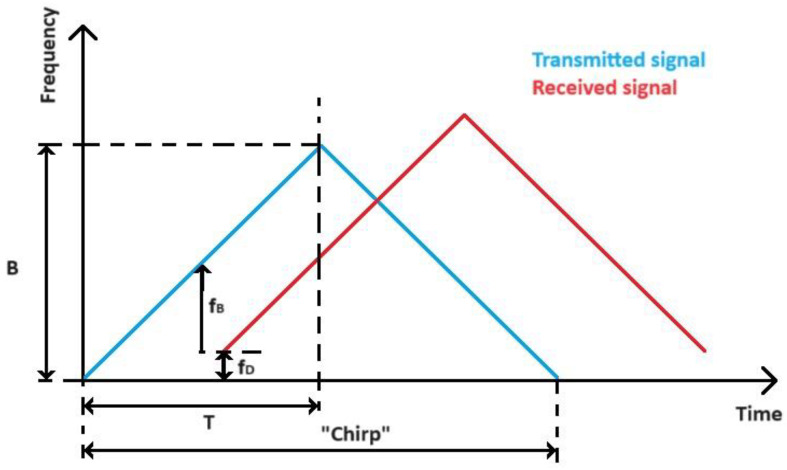
Chirp signal generated by FMCW radar.

The signal transmitted by the FMCW radar is called a chirp. It is assumed that the radar transmits a finite number of *K* chirps, each with a linearly increasing frequency as a function of time. The Figure 2 below shows a model of the chirp signal generated by the FMCW radar and labels its most important parameters.

The time dependence of the frequency of the emitted signal is shown by the following relationship [24]:

(1) $$f(t)=\beta t^{\prime }$$

where $\beta =\frac{B}{T}$ is the frequency slope; *T* denotes the chirp duration; B denotes the frequency bandwidth swept; and $t^{\prime }\;\in \left[0,T\right]$ and k=0,…,K−1.

The signal transmitted by the FMCW radar can be modeled as follows [24]:

(2) $$s(t)=\cos\left(2\pi f_{C}t+\varphi \left(t\right)\right)$$

where the time *t* and phase $\varphi \left(t\right)$ can be expressed as follows [24]:

(3) $$t=kT+t^{\prime }$$

(4) $$\varphi \left(t\right)=\pi k\beta T^{2}+\pi \beta {t^{\prime }}^{2}$$

**Figure 3:**
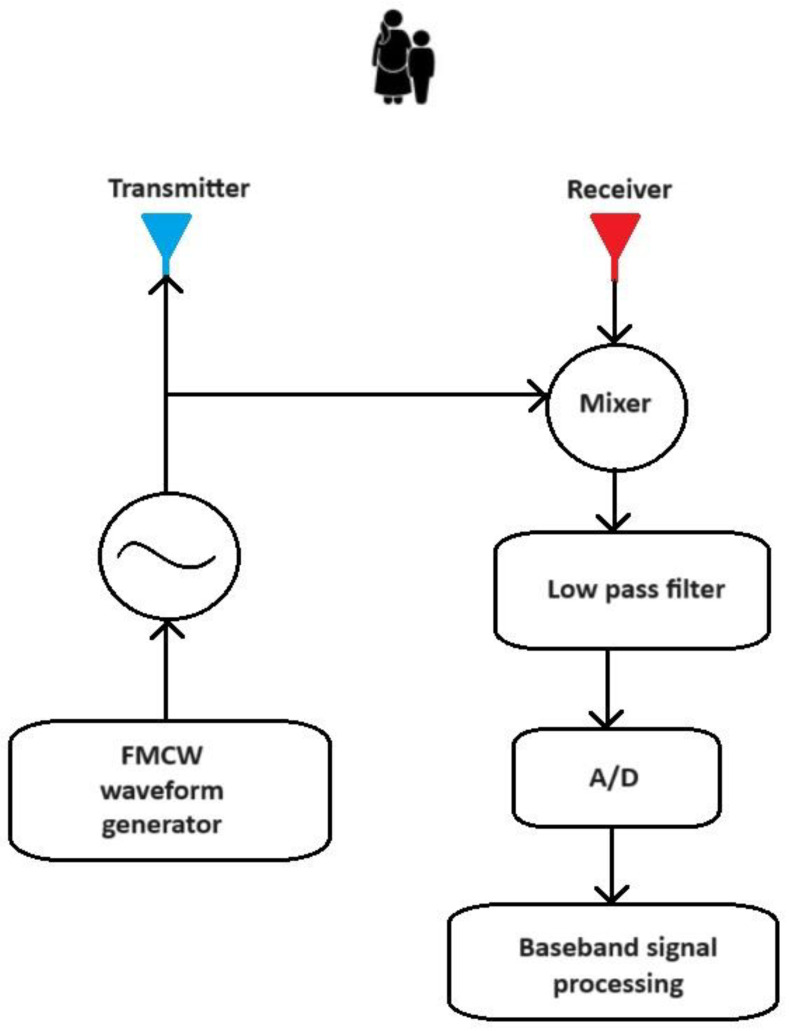
Block diagram of FMCW radar.

If the transmitted signal encounters an object in its path, part of the energy of the electromagnetic wave will return back to the radar. In the receiving part of the FMCW radar, the operation of multiplying the received signal with a replica of the transmitted signal and filtering takes place, which ultimately results in a signal at the output of the receiving path, the form of which can be expressed as follows [24]:

(5) $$y(t)\approx \varepsilon e^{-j2\pi f_{B}t^{\prime }}e^{-j2\pi f_{D}kT}$$

where ε denotes the complex coefficient depending on antenna gain; $f_{B}$ denotes the beat frequency; and $f_{D}$ denotes the Doppler frequency. The situation described is shown in the Figure 3 below.

Knowing the values of $f_{B}$ and $f_{D}$, one can determine the velocity and distance to the object, respectively [25,26,27]:

(6) $$V=\frac{f_{D}c}{2f_{c}}$$

(7) $$R=\frac{f_{B}c}{2\beta }$$

where *c* denotes the speed of light.

### 3.2. Data Generation

**Figure 4:**
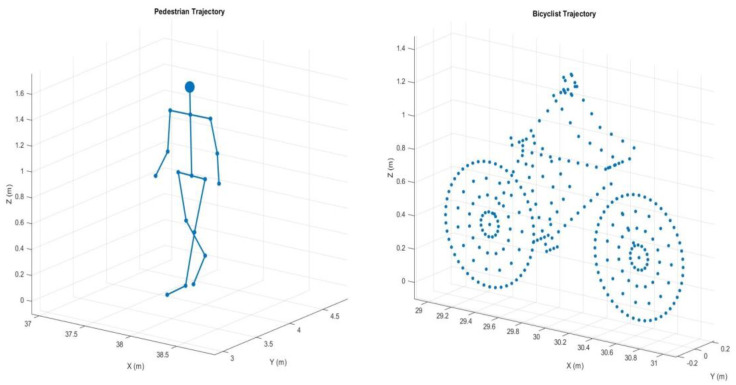
Simulated models of pedestrian and cyclist.

The input data for the neural network were generated in the Matlab (R2023b) environment using built-in functions that simulate the movement of a pedestrian, a cyclist, and a car [27]. This paragraph will describe the various properties and parameters set for each object model. Finally, the scheme of radar data generation and processing will be presented, up to the final classification of the object recognized by the system. Figure 4 shows the three-dimensional model of the pedestrian and cyclist used in this article.

The human model consists of 16 body segments, that is the neck and head, left and right shoulder, left and right upper limb, left and right lower arm, left and right upper arm, left and right hip, left and right lower limb, left and right foot, with echoes from the pedestrian including components from each segment. The three-dimensional initial location, random pedestrian height, random constant walking speed, and pedestrian turn and direction were assumed for the pedestrian gait parameters.

The model of the cyclist actually consists of the rider and the bicycle. Hence, the cyclist model consists of seven segments, which include the frame of the bicycle and the cyclist, the pedals of the bicycle, the upper and lower legs of the cyclist, the rear wheel, and the front wheel of the cyclist. The parameters of the cyclist include a three-dimensional initial location, a random constant speed of movement, the turn and direction of movement, the number of spokes in the bicycle wheel, the ratio of wheel rotation to pedal rotation, and information about whether the cyclist is pedaling or not pedaling during the simulation.

The car model was described as an object that is a specified platform in the Matlab environment. Hence, a three-dimensional initial location, a random constant displacement speed, turn and direction of displacement, and a specified value of effective reflective area were assumed for the car’s parameters.

**Figure 5:**
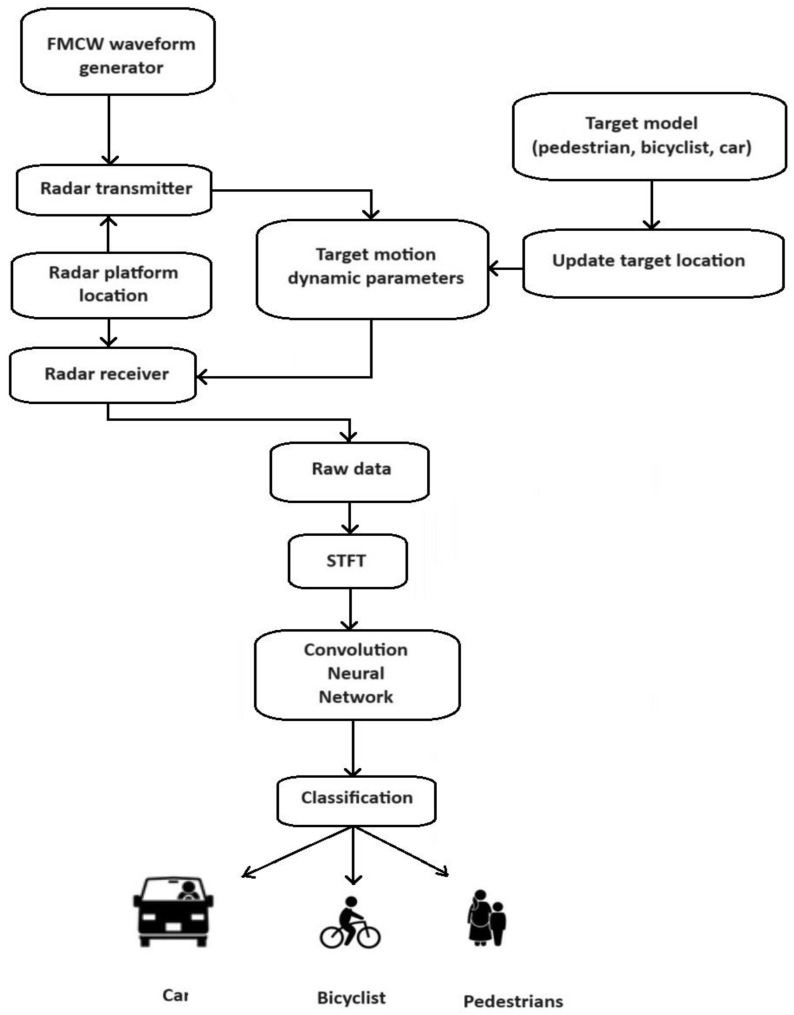
Block diagram of simulation.

This article proposes a novel method of generating input data for a convolutional neural network. Its scheme is shown in Figure 5. In the first step, a signal is generated by the FMCW radar. The signal goes to the transmitter, which emits the signal into space. A specific area in front of the radar was assumed, in which the pedestrian, cyclist, and car models described earlier were placed. This article assumes 10 cases of different configurations of objects in the radar beam. Part of the signal emitted by the radar returns back to the radar receiver, where further processing of the received signal takes place.

In the case of a non-rigid pedestrian target, in addition to the pedestrian’s actual movement (as the center of gravity), there are usually micro-movements, such as waving of the limbs or turning of the head. The effect of Doppler frequency modulation on radar echoes caused by micro-movements is referred to as the micro-Doppler effect.

The micro-Doppler can be visually plotted in the form of a spectrogram using the short-time Fourier transform (STFT); hence, the received signal in the radar’s receiving path was subjected to the Fourier transform (STFT). Even if the pedestrian’s center of gravity is moving at a constant speed, different parts of the human body can have different speeds, as well as acceleration and deceleration profiles. Therefore, the spectrogram of a radar echo from a walking pedestrian will contain different Doppler signatures produced by different parts of the body. The speed and periodicity information associated with walking can be used as unique radar signatures to detect and classify pedestrians.

The spectrograms obtained in the above way for 10 scenarios form the input data for a special convolutional neural network structure that is the final classifier distinguishing between the types of objects in front of the radar. The 10 cases of occurrence of different objects in front of the radar were assumed for the simulation:

1. Single pedestrian;
2. Two pedestrians;
3. Three pedestrians;
4. Single cyclist;
5. Single car;
6. Single pedestrian and single car;
7. Single pedestrian and single cyclist;
8. Single car and single cyclist;
9. Two pedestrians and a single car;
10. Two pedestrians and a single cyclist.

**Figure 6:**
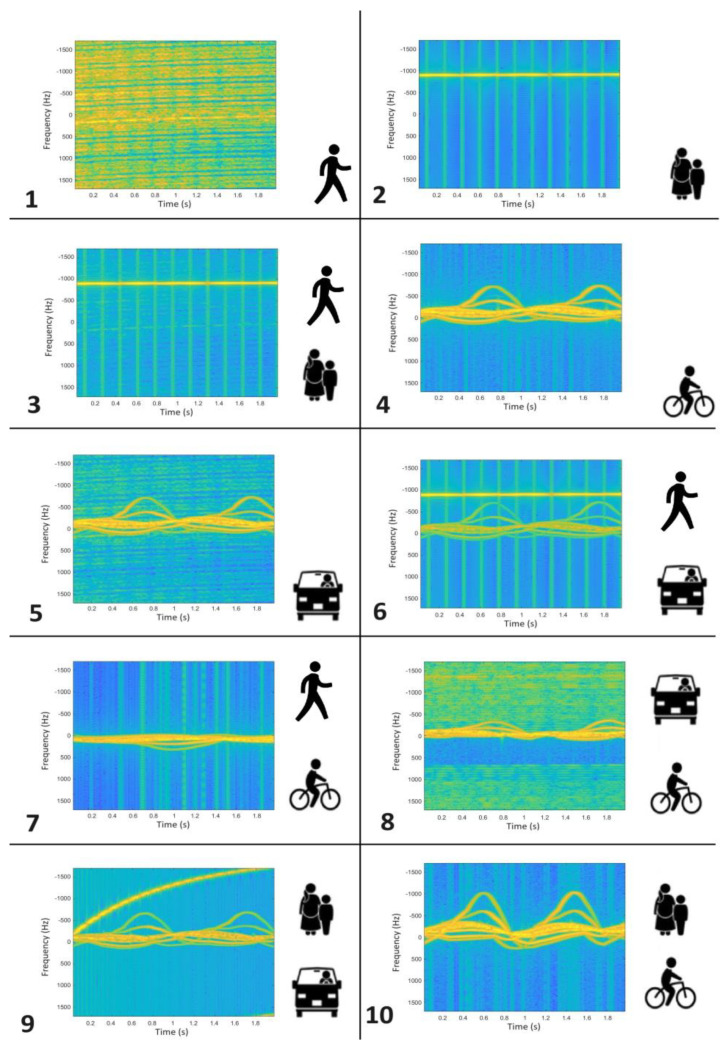
Datasets: 1−single pedestrian; 2−two pedestrians; 3−three pedestrians; 4−single cyclist; 5−single car; 6−single pedestrian and single car; 7−single pedestrian and single cyclist; 8−single car and single cyclist; 9−two pedestrians and a single car; 10−two pedestrians and a single cyclist.

The image database contained 1000 images for each of the 10 classes, each image having a dimension of 875 × 656. Examples of images representing all 10 classes are shown in Figure 6. Considering the representations of all classes, one should find great difficulty in unambiguously determining the membership of a given image in the correct class. As can be seen, the image database is heavily imbalanced in terms of the proportion of each class, hence, it is difficult to process. This is another problem that the authors have solved in this article.

This article uses radar imagery generated in the Matlab environment. Nevertheless, it is worth noting that there are many databases with radar imagery widely available on the Internet, freely available for scientific research purposes [7]. For the authors, the purpose of this article is to test the performance and correctness of the proposed convolutional neural network structure for generated radar signals reflected from selected available objects.

### 3.3. The Short-Time Fourier Transform

This paragraph discusses the basics of the time-varying frequency analysis of non-stationary signals using the short-time Fourier transform (STFT). The application of the STFT applies to all measurement situations in which it is important to extract the information contained in the time- or space-varying frequency spectrum of the signal under study. The continuous short-time Fourier transform (STFT) can be interpreted as being an undiscretized-in-time-and-frequency Gabor transform. The definition of this transformation in the time and frequency domain is as follows [28]:

(8) $${STFT}_{x}^{T}\left(t,\;f\right)=\int_{-\infty }^{+\infty }x\left(\tau \right)\gamma^{*}\left(\tau -t\right)e^{-j2\pi ft}d\tau$$

(9) $${STFT}_{x}^{F}\left(t,\;f\right)=e^{-j2\pi ft}\int_{-\infty }^{+\infty }X\left(\upsilon \right)\Gamma^{*}\left(\upsilon -f\right)e^{j2\pi \upsilon t}d\upsilon$$

where the function $\gamma^{\;}\left(t\right)$ denotes the temporal observation window; and $\Gamma^{\;}\left(f\right)$ is its Fourier spectrum. Equations (8) and (9) are often called “the Moving Window Method (MWM)” in the time domain (8) and frequency domain (9), respectively. In the time domain, STFT involves performing a simple Fourier transform on successive signal fragments, “clipped” by a moving window $\gamma^{\;}\left(t\right)$. For the short-time Fourier transform, the so-called spectrogram is defined as the square of its modulus [28]:

(10) $$S_{x}^{SPECTOGRAM}\left(t,\;f\right)={\left|{STFT}_{x}^{\;}\left(t,\;f\right)\right|}^{2}$$

### 3.4. Deep Learning Network

This article uses the CNN model, which is characterized by a multilayer (deep) structure. The model includes inter-area convolutional connections in the first layers, which allows for efficient feature extraction from input data. Batch normalization layers were also used during the learning process to speed up the learning process and stabilize the network [29]. The model also uses a linking layer, which contributes to reducing the dimensionality of the data and increasing the invariance to translation [30].

**Figure 7:**
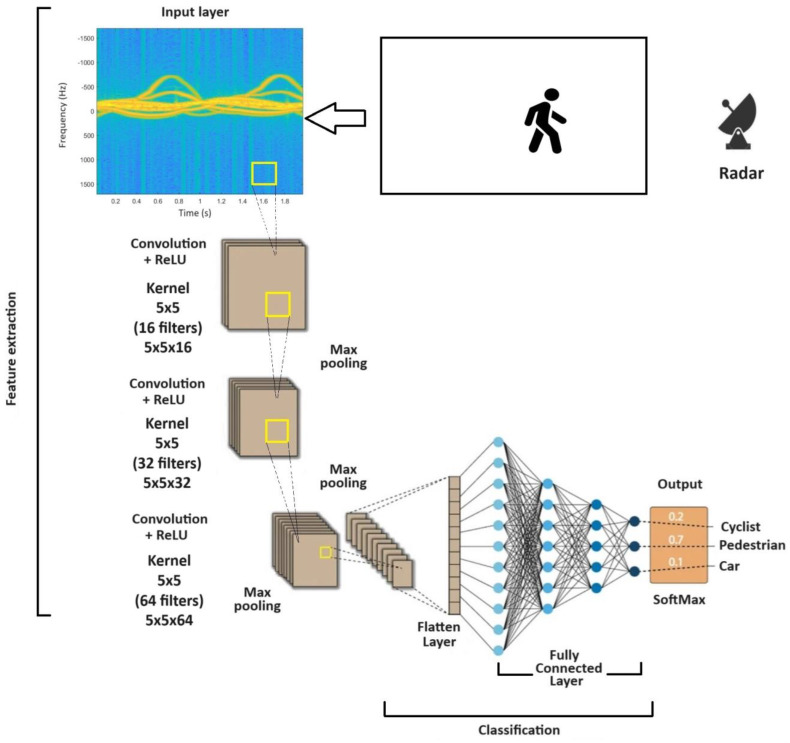
Data flow structure and CNN network structure.

These elements make up the comprehensive architecture of the CNN model, which enables efficient classification of input data. The architecture of the network used is shown in Figure 7.

The CNN network was created from scratch using a program in Matlab. The final network structure, defined after many preliminary experiments, contained four convolutional layers:

1. The first layer consists of 16 neurons, 5 × 5 filter reception field, 2 × 2 stride, batch normalization layer, ReLU, max pooling;
2. The second layer is composed of 32 neurons, 5 × 5 filter reception field, stride 2 × 2, batch normalization layer, ReLU, max pooling;
3. The third layer is composed of 64 neurons, 5 × 5 filter reception field, stride 2 × 2, batch normalization layer, ReLU, max pooling;
4. The fourth layer is composed of 128 neurons, 5 × 5 filter reception field, stride 2 × 2, batch normalization layer, ReLU, max pooling.

In addition, there was a flatten layer, one fully connected layer containing 10 neurons representing 10 recognized image classes.

The input layer of a convolutional neural network is the first layer of the network that accepts input data. The input layer does not perform any convolution or activation operations. Its task is only to accept the input data and pass it to subsequent layers of the network, preserving its original dimensions and properties. This is the starting point of the neural network’s data processing [31].

The convolutional layer is a key element in convolutional neural networks (CNNs). The convolution operation is a basic image processing technique that allows feature extraction from input data by moving a filter (mask) over the image and calculating the scalar product between image pixel values and filter weights [31]. In the case of images, the data array is represented by a two-dimensional matrix *I* with elements representing pixel brightness degrees *I*(*m*, *n*), and the kernel *K* is two-dimensional. The two-dimensional spline operation is then written in the form [32]:

(11) $$Y\left(i,j\right)=I\left(i,j\right)*K\left(i,j\right)$$

(12) $$Y\left(i,j\right)=\sum_{m}\sum_{n}I\left(m,n\right)K\left(i-m,\;j-n\right)=\sum_{m}\sum_{n}I(i-m,j-n)K(m,n)$$

In CNNs, the input for the first hidden layer is an RGB representation of images set in tensor form. The convolution operation in the first convolutional layer includes all three RGB channels of the image (the sum of the individual channels), each with different assumed values of the kernel weights in the form of a linear filter constituting the analysis neuron. In further layers, this operation includes multiple (user-determined) images from the preceding layer.

In data processing, the convolution operation used in CNNs is distinguished by important advantages over ordinary matrix operations in classical networks. These include the locality of the connections, the common (repeatable) values of the connection weights of the image-shifting filter, and the resulting invariance (equivariance) with respect to the shift [32].

Batch normalization is used to combat a phenomenon known as internal covariate shift of distribution; this is when the distribution of input data to individual layers changes during training, making the network learning process more difficult. Batch normalization works by normalizing the input data for each training mini-patch (batch). This involves calculating the mean and standard deviation for each feature in a given mini-batch, and then transforming the input data so that they have a mean of zero and a unit standard deviation. In this way, even when the model parameters change during training, the input data are kept within a stable range of values, which facilitates the network learning process [33].

## 4. Experiments and Results

Based on the data in the tables below, simulations were carried out to teach a deep neural network capable of recognizing 10 classes of objects. Table 2 contains the parameters of the FMCW radar used for signal generation. Table 3 contains the parameters of the pedestrian, cyclist, and car movement.

In order to carry out the research, the following equipment (hardware and software) was used:

- Dell 13th Gen Intel(R) Core(TM) i5-1345U 1.60 GHz, 16 GB RAM, Windows 11 Pro;
- Matlab Version: 23.2.0.2365128 (R2023b), manufacturer: MathWorks, Inc., Natick, MA, USA.

The research experiment was conducted on 10,000 images (10 object classes of 1000 images each). The images were randomly divided into three sets of data: training data (70% of images) were used to estimate the weights of the artificial neural network, validation data (15% of images) were used to test the trained network, and test data (15% of images) were used to test the functioning of the network after training.

**Figure 8:**
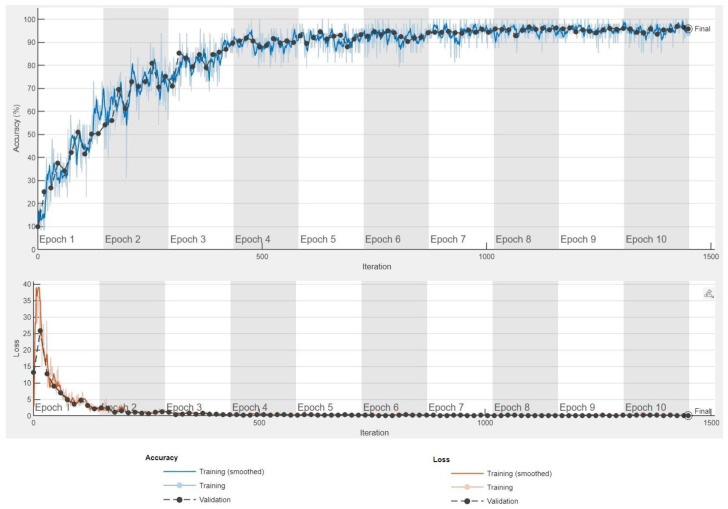
Training progress.

**Figure 9:**
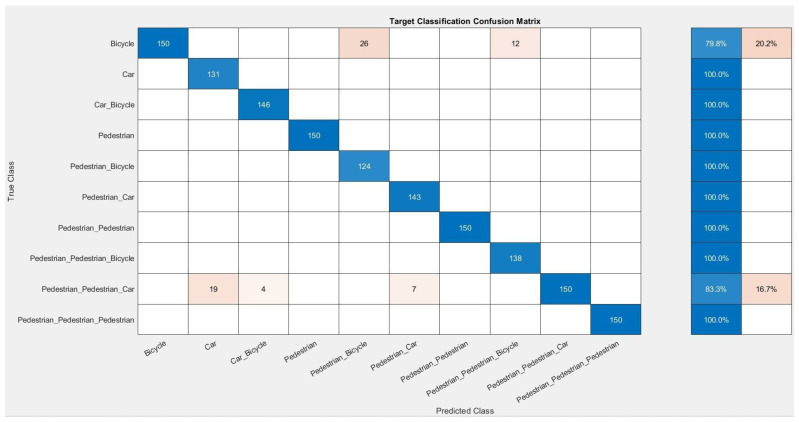
Confusion matrix.

Figure 8 shows the simulation results in the form of a learning curve for 10 epochs, where the accuracy reached 95.93%. In addition, a confusion matrix is presented in Figure 9 to show how the adopted model performs against target classes in the dataset.

The confusion matrix shows display examples that were properly classified (blue) against misclassified examples (orange). As can be seen, the adopted model made errors when clearly distinguishing between a single cyclist and the case where there were two pedestrians and a car in front of the radar.

The simulation results presented above are the best of the many results obtained. They are the result of time-consuming simulations that differed in many particular parameters such as the number of images for each class, the number of epochs, or the different values of the neural network hyperparameters. A summary of all simulations performed is presented collectively in Table 4.

**Figure 10:**
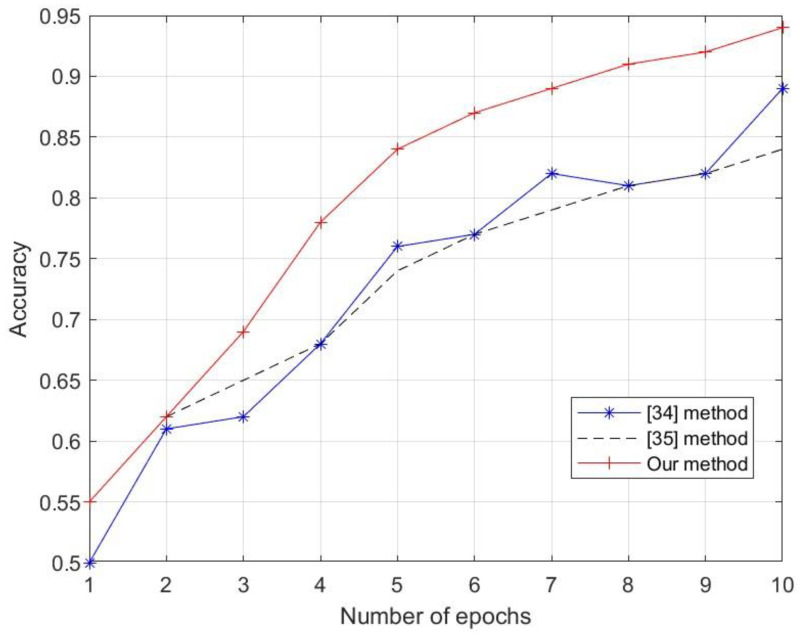
Comparison of three object recognition methods.

In order to check the performance of the proposed method, a comparison was made with other available methods. For this purpose, the methods described in the articles [34,35] were implemented in the Matlab environment. The first paper formulates the multiuser automatic modulation classification (mAMC) of compound signals as a multi-label learning task, which aims to recognize the modulation type of each constituent signal in a compound signal [34]. The second article proposes a semantic-based learning network (SLN) that simultaneously learns modulation classification and parameter regression of frequency-modulated continuous-wave (FMCW) signals [35]. In order to use common input data, modifications were made to both of the above methods so that they were capable of recognizing 10 classes of objects. Figure 10 shows the performance of the three methods, capable of recognizing pedestrians, cyclists, and cars. The research experiment was conducted on 10,000 images (10 object classes of 1000 images each). The images were randomly divided into three sets of data: training data (70% of images) were used to estimate the weights of the artificial neural network, validation data (15% of images) were used to test the trained network, and test data (15% of images) were used to test the functioning of the network after training.

The results obtained suggest the highest accuracy for the method proposed by the authors of this article. Some attention should be paid to the obtained results. The selection of a neural network structure is never random. According to the literature, it has been shown that choosing a reasonable structure at the beginning, well-suited to the specifics of the task to be solved, can significantly reduce the learning time and improve the final results [36]. Hence, the authors conclude that one of the reasons for the best results for the newly developed method is the selection of the right network structure and learning process for the input data.

## 5. Discussion and Conclusions

Deep neural networks and related deep learning have created new prospects for the development of artificial intelligence, while the creative combination of this technology with radar allows their real application in the daily functioning of ordinary people.

Deep neural networks, which combine simultaneously two functions—that is, the generation of diagnostic features and the final classifier—are able to process original data without the need for preliminary expert analysis.

After a deep analysis of the literature, the authors of this article proposed a missing approach based on simulated data generated in the Matlab environment and a novel method of identifying objects using an FMCW radar operating at 77 GHz, processing the obtained signals using STFT, and then the obtained spectrograms using a specialized deep neural network structure.

Accordingly, this paper presents a method for recognizing and distinguishing a group of objects on the basis of radar signatures of objects and a special convolutional neural network structure. The proposed approach is based on a database of radar signatures generated on pedestrian, cyclist, and car models in the Matlab environment.

Innovative aspects of the work include a method for discriminating multiple objects on a single radar signature, a dedicated convolutional neural network architecture, and the use of a method for generating a custom input database.

Section 4, which presents research experiments and recognition results for 10 classes of objects, confirms the high efficiency of the developed method. In order to check the performance of the proposed method, a comparison was made with other available methods. For this purpose, the methods described in articles [34,35] were implemented in the Matlab environment. The results obtained suggest the highest accuracy for the method proposed by the authors of this article.

The obtained results of simulations and positive tests provide a basis for the application of this system in many sectors and areas of the economy. To be mentioned here is the possibility of realizing an intelligent vehicle that provides real-time pedestrian detection and warning mechanisms by vibrating the steering wheel and displaying a message on the monitor/dashboard to warn the driver of a collision with a pedestrian. In addition, the proposed solution can be used as an automatic detection system for people, animals, or vehicles, and can be used in sectors of the economy such as production halls where accidental uncontrolled interference by a person or other object can disrupt the normal operation of the enterprise or expose people, including employees, to loss of life or health.

Further research directions and work of the authors of this article will be directed towards the practical implementation of the above-mentioned research, including the purchase of the necessary FMCW radar, the execution of practical tests, and the verification and improvement of the developed convolutional neural network structure.

## Figures and Tables

**Table 1:** List of acronyms.

Acronym	Definition
ATR	Automatic Target Recognition
CNN	Convolutional Neural Network
DBN	Deep Belief Network
DL	Deep Learning
FMCW	Frequency-Modulated Continuous-Wave
HRRP	High-Resolution Range Profiles
LSTM	Long Short-Term Memory
MWM	Moving Window Method
RBM	Restricted Boltzmann Machine
SCF	Spectral Signature Patterns
STFT	Short-Time Fourier Transform

**Table 2:** Parameters of radar FMCW.

Parameter	Value
Carrier frequency	77 GHz
Range resolution	0.5 m
Maximum detectable range	100 m
Maximum detectable speed	50 km/h
Sweep time	3.667 ms
Sample rate	300 MHz
Maximum beat frequency	54.55 MHz
Maximum Doppler shift	7.13 kHz

**Table 3:** Parameters of the pedestrian, cyclist, and car.

Pedestrian	
**Parameter**	**Value**
Initial position	$x\epsilon \left[0,\;50\right]\;$ $y\epsilon \left[-10,\;10\right]$ z=0
RCS	[0.5, 1] m^2^
Height	$\left[1.5,\;2.0\right]\;m$
Speed	$\left[0.1\;,\;1.0\right]\;m/s$
Heading	$\left[0,\;360\right]\;\mathrm{degree}$
**Cyclist**	
**Parameter**	**Value**
Initial position	$x\epsilon \left[0,\;50\right]\;$ $y\epsilon \left[-10,\;10\right]$ z=0
RCS	[2, 4] m^2^
Number of spokes per wheel	18
Speed	$\left[1,\;10\right]\;m/s$
Heading	$\left[0,\;360\right]\;\mathrm{degree}$
Ratio of wheel rotations to pedal rotations	$\left[0.5,\;6\right]$
Pedaling status information	True or False
**Car**	
**Parameter**	**Value**
Initial position	$x\epsilon \left[0,\;50\right]\;$$y\epsilon \left[-10,\;10\right]$ z=0
RCS	[10, 20] m^2^
Height	$\left[1.5,\;2.0\right]\;m$
Motion model	Constant velocity
Speed	$\left[0.1,\;1.0\right]\;m/s$
Radar cross-section	10 m^2^

**Table 4:** Summary of simulation results.

Simulation 1	
**Number of images (per class)**	**Accuracy**
50	33.23%
100	56.67%
400	71.08%
700	88.54%
1000	94.22%
**Simulation 2 (10 classes of 1000 images each)**	
**Number of epochs**	**Accuracy**
3	88.54%
5	92.12%
7	94.04%
10	95.22%
**Simulation 3 (10 classes of 1000 images each, 10 epochs)**	
**Initial learning rate**	**Training time**
0.00001	2560 min
0.0001	1140 min
0.001	450 min

## Data Availability

The data presented in this study are available upon request from the corresponding author.

## References

[1] Schmidhuber J. (2015). Deep learning in neural networks: An overview. Neural Netw..

[2] Goodfellow I., Bengio Y., Courville A. (2016). Deep Learning.

[3] Fukushima K. (1980). Neocognitron—A self-organizing neural network model for a mechanism of pattern recognition unaffected by shift in position. Biol. Cybern..

[4] LeCun Y., Bengio Y. (1995). Convolutional Networks for Images, Speech, and Time-Series. The Handbook of Brain Theory and Neural Networks.

[5] Krizhevsky A., Sutskever I., Hinton G.E. (2012). Imagenet classification with deep convolutional neural networks. Adv. Neural Inf. Process. Syst..

[6] Hinton G.E., Osindero S., Teh Y.W. (2006). A fast learning algorithm for deep belief nets. Neural Comput..

[7] Geng Z., Yan H., Zhang J., Zhu D. (2021). Deep-Learning for Radar: A Survey. IEEE Access.

[8] Rypulak A. (2023). Sensory obrazowe bezzałogowych statków powietrznych.

[9] Mason E., Yonel B., Yazici B. Deep learning for radar. Proceedings of the 2017 IEEE Radar Conference (RadarConf).

[10] Chen V. (2019). The Micro-Doppler Effect in Radar.

[11] Ying L., Chen Y., Zhu Y., Li W., Zhang Q. (2020). Doppler effect and micro-Doppler effect of vortex-electromagnetic-wave-based radar. IET Radar Sonar Navig..

[12] Baczyk M.K., Samczynski P., Kulpa K., Misiurewicz J. (2015). Micro-Doppler signatures of helicopters in multistatic passive radars. IET Radar Sonar Navig..

[13] Belgiovane D., Chen C. Micro-Doppler characteristics of pedestrians and bicycles for automotive radar sensors at 77 GHz. Proceedings of the 2017 11th European Conference on Antennas and Propagation (EUCAP).

[14] Dadon Y., Yamin S., Feintuch S., Permuter H., Bilik I., Taberkian J. Moving target classification based on micro-Doppler signatures via deep learning. Proceedings of the IEEE Radar Conf. (RadarConf).

[15] Yang Y., Hou C., Lang Y., Sakamoto T., He Y., Xiang W. (2020). Omnidirectional motion classification with monostatic radar system using microDoppler signatures. IEEE Trans. Geosci. Remote Sens..

[16] Hadhrami E.A., Mufti M.A., Taha B., Werghi N. Ground Moving Radar Targets Classification Based on Spectrogram Images Using Convolutional Neural Networks. Proceedings of the 19th International Radar Symposium (IRS).

[17] Hadhrami E.A., Mufti M.A., Taha B., Werghi N. Transfer learning with convolutional neural networks for moving target classification with micro-Doppler radar spectrograms. Proceedings of the International Conference on Artificial Intelligence and Big Data (ICAIBD).

[18] Hadhrami E.A., Mufti M.A., Taha B., Werghi N. (2019). Learned Micro-Doppler Representations for Targets Classification Based on Spectrogram Images. IEEE Access.

[19] Kim B.K., Kang H.-S., Park S.-O. (2017). Drone Classification Using Convolutional Neural Networks With Merged Doppler Images. IEEE Geosci. Remote Sens. Lett..

[20] Rahman S., Robertson D. (2020). Classification of drones and birds using convolutional neural networks applied to radar micro-Doppler spectrogram images. IET Radar Sonar Navig..

[21] Mendis G.J., Wei J., Madanayake A. Deep learning cognitive radar for micro UAS detection and classification. Proceedings of the Cognitive Communications for Aerospace Applications Workshop (CCAA).

[22] Mendis G.J., Randeny T., Wei J., Madanayake A. Deep learning based doppler radar for micro UAS detection and classification. Proceedings of the MILCOM 2016—2016 IEEE Military Communications Conference.

[23] Feng B., Chen B., Liu H. (2017). Radar HRRP target recognition with deep networks. Pattern Recognit..

[24] Jankiraman M. (2018). FMCW Radar Design.

[25] Skolnik M. (2008). Radar Handbook.

[26] Budge M., German S. (2020). Basic Radar Analysis.

[27] Cuevas E., Luque A., Escobar H. (2024). Computational Methods with MATLAB.

[28] Grigoryan A.M. (2005). Fourier transform representation by frequency-time wavelets. IEEE Trans. Signal Process..

[29] Giusti A., Cireşan D.C., Masci J., Gambardella L.M., Schmidhuber J. Fast image scanning with deep max-pooling convolutional neural networks. Proceedings of the 2013 IEEE International Conference on Image Processing.

[30] Agarwal T., Sugavanam N., Ertin E. Sparse Signal Models for Data Augmentation in Deep Learning ATR. Proceedings of the 2020 IEEE Radar Conference (RadarConf20).

[31] Matuszewski J., Pietrow D. (2021). Specific Radar Recognition Based on Characteristics of Emitted Radio Waveforms Using Convolutional Neural Networks. Sensors.

[32] Osowski S. (2020). Sieci neuronowe do przetwarzania informacji.

[33] Loffe S., Szegedy C. Batch normalization: Accelerating deep network training by reducing internal covariate shift. Proceedings of the 32nd International Conference on Machine Learning.

[34] Mengtao Z., Yunjie L., Zesi P., Jian Y. (2020). Automatic modulation recognition of compound signals using a deep multi-label classifier: A case study with radar jamming signals. Signal Process..

[35] Chen K., Zhang J., Chen S., Zhang S., Zhao H. (2023). Recognition and Estimation for Frequency-Modulated Continuous-Wave Radars in Unknown and Complex Spectrum Environments. IEEE Trans. Aerosp. Electron. Syst..

[36] Tadeusiewicz R., Gąciarz T., Borowik B., Leper B. (2007). Odkrywanie właściwości sieci neuronowych przy użyciu programów w języku C#.

